# Inverted-U association between daily steps and WHO-5 in university students: non-linear modeling and robustness checks

**DOI:** 10.3389/fnbeh.2025.1693386

**Published:** 2025-10-24

**Authors:** Huakai Zhang, Shiguang Wang, Yongchao Huang, Lei Xiu, Yan Wang

**Affiliations:** ^1^Medical College, Zhengzhou University of Industrial Technology, Zhengzhou, Henan, China; ^2^Henan Province Smart Health Care Engineering Technology Research Center, Zhengzhou, Henan, China

**Keywords:** subjective wellbeing, daily step count, dose–response, non-linear association, university students, sleep quality, perceived stress

## Abstract

**Background:**

Physical activity is linked to mental health, yet the dose–response shape remains debated.

**Methods:**

In a cross-sectional sample of Chinese university students, 820 participants (mean age 21.5 years; 51.8% women) wore wrist accelerometers for 7 days. Subjective well-being (SWB) was measured with the WHO-5 (0–100). Restricted cubic spline models adjusted for age, sex, sleep quality, perceived stress, and socioeconomic status. Sensitivity analyses included quadratic and segmented models, trimming/winsorization, and E-value assessment. Peaks/plateaus were estimated via the delta method and bootstrap-BCa confidence intervals.

**Results:**

The steps–SWB association was non-linear (overall p<0.05). SWB rose steeply up to ~8,650 steps/day and then leveled off, with a statistical plateau near ~19,300 steps/day (bootstrap-BCa 95% CI: 7,997–17,896; delta-method 95% CI: 9,394–14,462). No contrast versus 4,000 steps/day exceeded the prespecified minimal clinically important difference (MCID=10 points). Findings were consistent across specifications; right-tail precision was limited due to few very high step counts.

**Conclusion:**

Among university students, higher daily steps are associated with better SWB up to ~8,000–12,000 steps/day, beyond which benefits plateau with diminishing returns rather than harm. Results support range-based, progressive step guidance for student mental health. Please replace the current abstract with the structured IMRaD version provided above.

## 1 Introduction

Subjective wellbeing (SWB) is a core indicator of mental health and quality of life. A large body of research links physical activity (PA) with higher SWB across age groups and across multiple facets of wellbeing (e.g., affective wellbeing and life satisfaction; Buecker et al., 2021; Li et al., 2023; Patria, 2022; Bernstein and McNally, 2017; Bernstein and McNally, 2018; Wang et al., 2025; Weinstein et al., 2024). Building on this literature, accumulating evidence suggests that the PA–wellbeing relationship is non-linear: benefits are greater within moderate levels of PA, with diminishing returns—and in some cases, a slight decline—beyond higher doses (Chekroud et al., 2018; Shimura et al., 2022).

For quantifying behavioral dose, the daily step count offers a low-biased, continuously scaled exposure. Recent systematic reviews and meta-analyses show dose–response associations between more daily steps and lower risk or symptoms of depression, with plateaus or attenuated gains often observed at approximately 7,000–10,000 steps/day, supporting the use of step counts for actionable recommendations (Bizzozero-Peroni et al., 2024; Inoue et al., 2023). Evidence on steps and multiple health outcomes—including mental health-related endpoints—has been growing rapidly, underscoring the advantages and comparability of device-measured steps (Ding et al., 2025).

University students represent a particularly important population for this line of inquiry: their daily routines are irregular, and academic and social pressures are salient (Haruna et al., 2025; Pérez-Jorge et al., 2025), and they are heavy users of wearable devices, making them both vulnerable to fluctuations in wellbeing and highly suitable for step-based monitoring (Abd-Alrazaq et al., 2024; Pope et al., 2019). Despite these features, little is known about the precise dose–response relationship between step counts and SWB in this group. Addressing this gap not only advances theory on non-linear PA–wellbeing links but also yields practical, range-based targets for campus health promotion (Bizzozero-Peroni et al., 2024; Ding et al., 2025).

Against this background, we investigated Chinese university students using wearable-recorded daily step count as exposure and WHO-5 as the outcome. We explicitly hypothesized that SWB would increase with step count up to a threshold, after which the association would plateau or modestly decline, forming an inverted-U pattern.

## 2 Materials and methods

### 2.1 Study design and participants

This cross-sectional observational study recruited university students from a campus in Zhengzhou, China, between 1 May 2025 and 1 July 2025.

The inclusion criteria were as follows: patients aged ≥18 years; those able to independently complete questionnaires and provide written informed consent; those who provided at least 7 consecutive days of wearable step-count data; and those who completed the World Health Organization-Five Well-Being Index (WHO-5), the Pittsburgh Sleep Quality Index (PSQI), and the Perceived Stress Scale-4 (PSS-4).

The exclusion criteria were as follows: those who were previously diagnosed with severe neurological or psychiatric disorders, those with missing or invalid step data (>30% missing), and the use of assistive devices that substantially affect gait.

Of the 1,050 students invited, 912 agreed to participate (86.9% responses). After excluding 92 with insufficient step-count device wear (<4 valid days) and 0 with incomplete questionnaires, 820 participants (78.1% of those invited) were retained for analysis.

All participants provided written informed consent. The study complied with the Declaration of Helsinki and was approved by the Institutional Review Board of Zhengzhou University of Industrial Technology (protocol code 202504036).

### 2.2 Measures and variable definitions

#### 2.2.1 Exposure: daily step count

Participants were wearing a HUAWEI Band 8 (Huawei Device Co., Ltd., Shenzhen, China) on the non-dominant wrist for 7 consecutive days. The device is equipped with six-axis inertial sensors (accelerometer and gyroscope) and an optical heart-rate sensor. We extracted daily step counts that were aggregated using the manufacturer’s algorithm via the Huawei Health application; however, raw accelerometry sampling frequencies are not publicly disclosed. Although the proprietary algorithm is not publicly available, validation studies have demonstrated acceptable step-count accuracy of Huawei wearables against research-grade accelerometers in free-living adults (Mei et al., 2024). A valid day was defined as ≥20 h of wear; participants with ≥4 valid days, including ≥1 weekend day, were retained for analysis.

#### 2.2.2 Primary outcome

Subjective wellbeing was measured using the Chinese WHO-5 Well-Being Index, which shows solid psychometric properties in Chinese university students and the general population (Fung et al., 2022; Du et al., 2023; Kliem et al., 2025). Each item is scored on a scale of 0–5; total scores were transformed to 0–100, with higher scores indicating better wellbeing. We adopted a minimal clinically important difference (MCID) of 10 points on the WHO-5 as a benchmark for interpreting the practical significance of observed associations.

#### 2.2.3 Covariates

We adjusted for a set of covariates that may plausibly influence subjective wellbeing (SWB). Age and sex were included as standard demographic factors. Sleep quality was assessed by the Pittsburgh Sleep Quality Index (PSQI) (De Moraes et al., 2024; Guo et al., 2016) and perceived stress by the 4-item Perceived Stress Scale (PSS-4) (She et al., 2021), both of which are established predictors of mental health. Monthly living expenses were included as a proxy indicator of socioeconomic status (SES). Because the distribution of expenses was highly right-skewed, we applied log-transformation to improve normality and model fit. We adjusted for age, sex, sleep quality, perceived stress, and SES because these are established correlates of SWB and likely confounders of the steps–SWB link (Blanchflower and Bryson, 2024; Wang et al., 2023; Jeong et al., 2024; Su and He, 2023).

Data auditing procedures included checks for timestamp continuity, abnormal spikes, and extended zero-count segments. Abnormal days were verified manually and corrected using pre-specified rules.

### 2.3 Statistical analysis

Analyses were conducted in R version 4.5.1. All models were adjusted for age, sex, sleep quality (PSQI), perceived stress (PSS-4), and monthly living expenses.

The primary dose–response model was specified as a restricted cubic spline (RCS) with four knots placed at the 5th, 35th, 65th, and 95th percentiles of mean daily steps. Overall non-linearity was assessed by a joint Wald/F-test of the spline terms (H₀: all non-linear terms = 0). For sensitivity analyses, we additionally fit quadratic polynomial models (testing *β*₂ = 0) and piecewise regression models with data-driven breakpoints.

Turning points were identified from the fitted RCS curve. To quantify uncertainty, we computed 95% confidence intervals (CIs) using both the delta method and 1,000 pairs of bootstrap replicates with bias-corrected and accelerated (BCa) intervals. We also evaluated whether the predicted differences in WHO-5 scores reached the MCID of 10 points, a benchmark commonly adopted for the WHO-5.

To assess robustness, we performed sensitivity analyses, including (i) winsorizing or truncating extreme step counts, (ii) alternative covariate specifications, and (iii) subgroup analyses.

To address unmeasured confounding, we calculated E-values for key step-count contrasts derived from the RCS model, quantifying the minimum strength of association an unmeasured confounder would need with both exposure and outcome to fully explain away the observed association.

To evaluate statistical adequacy, we further conducted a *post-hoc* power analysis for the overall non-linearity test in the primary RCS model. Power was estimated at *α* = 0.05 using the non-central-F distribution parameterized by the partial *R*^2^ of the non-linear spline terms.

## 3 Results

### 3.1 Sample characteristics by step-count quartiles

Among the 820 participants included, 336 (41.0%) contributed 7 valid days, 262 (32.0%) contributed 6 days, 146 (17.8%) contributed 5 days, and 76 (9.3%) contributed 4 days. Overall, 598 of the 820 participants (72.9%) provided ≥6 valid days.

A total of 820 participants (mean age 21.5 ± 2.1 years, 51.8% women) were included in the analysis. The average sleep quality score (PSQI) was 6.3 ± 2.6, and the perceived stress score (PSS-4) was 6.9 ± 3.2. The median monthly living expense was CNY¥2,242 [IQR: 1,714–2,957]. Device compliance was high, with participants contributing a median of 7 valid days [IQR: 5–9] and an average wear time of 21.6 ± 0.36 h/day.

By design, mean daily steps increased across quartiles: 5,513 ± 1,474 in Q1, 8,422 ± 586 in Q2, 10,519 ± 592 in Q3, and 13,209 ± 1,428 in Q4. Baseline characteristics such as sex, age, sleep, stress, and living expenses were well balanced across step-count quartiles (all |SMD| < 0.10). Detailed characteristics are provided in Supplementary Table 1.

### 3.2 Main dose–response

The RCS model demonstrated a statistically significant overall non-linearity in the association between mean daily steps and WHO-5 scores (*p* for non-linearity < 0.05; Supplementary Table 2).

**Figure 1:**
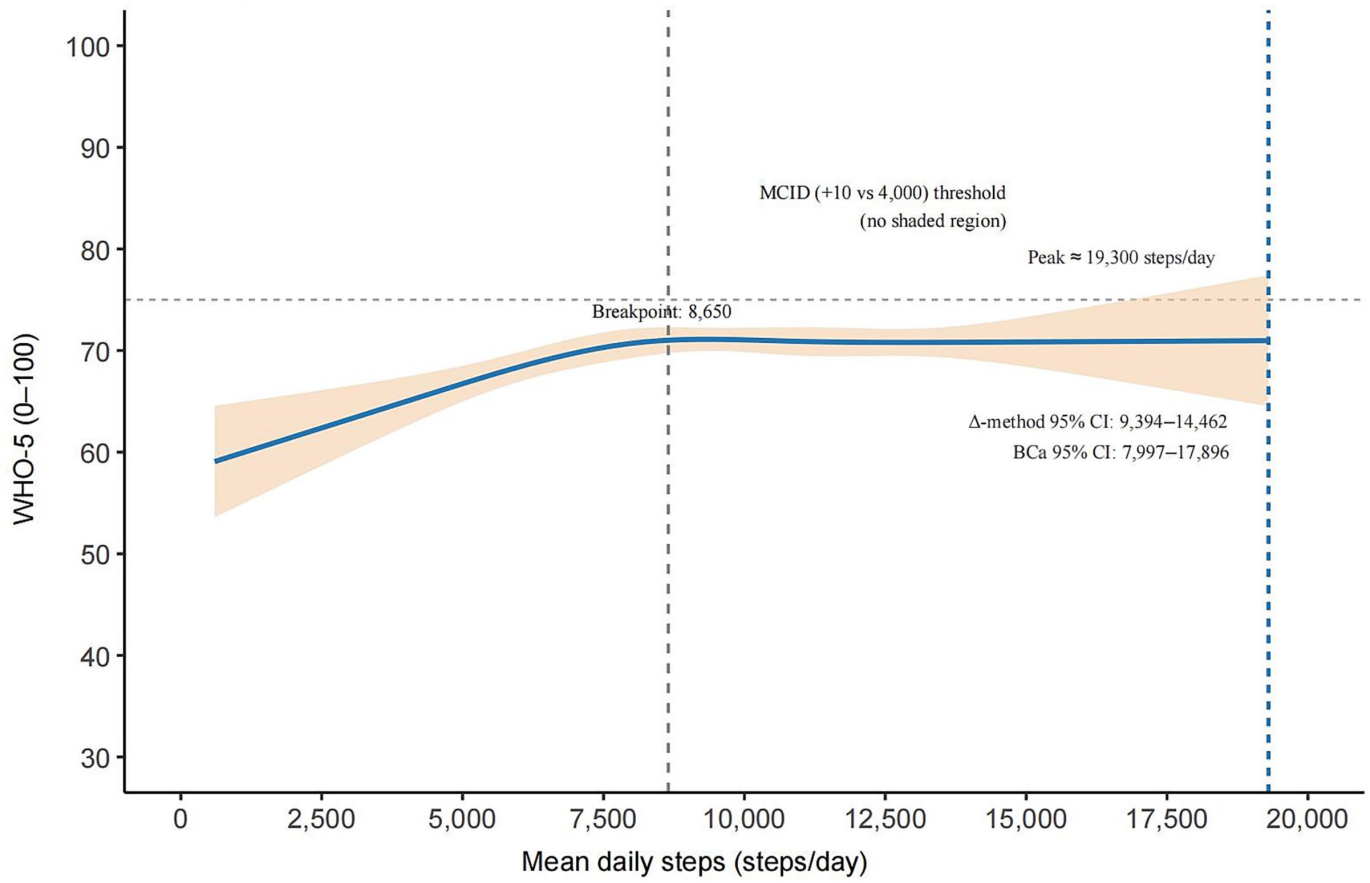
Adjusted non-linear dose–response between mean daily steps and WHO-5 in university students (RCS). The solid blue curve shows the adjusted association with a 95% robust CI (shaded). The vertical dashed line marks the maximum of the fitted curve (peak ≈ 19,300 steps/day; BCa 95% CI 7,997–17,896; *Δ*-method 95% CI 9,394–14,462). The dotted gray line indicates the segmented regression breakpoint (~8,650 steps/day). Relative to 4,000 steps/day, no step-count range achieved the minimal clinically important difference (MCID = +10 points); hence, the MCID threshold is shown as a horizontal reference line (no shaded region).

In the RCS model (Figure 1), WHO-5 scores increased with step count, showing a steep increase up to approximately ~8,650 steps/day and a gradual leveling thereafter. The curve reached a plateau at approximately ~19,300 steps/day (bootstrap-BCa 95% CI: 7,997–17,896; delta method 95% CI: 9,394–14,462), without the evidence of a clinically meaningful decline at higher levels.

Although the dose–response was statistically significant, the maximum contrast in predicted WHO-5 scores relative to 4,000 steps/day did not exceed the prespecified minimal clinically important difference (MCID) of 10 points, indicating that the magnitude of the change may be of limited clinical relevance.

Model comparison results (Supplementary Table 3) further supported the RCS specification, which provided a better fit than linear or quadratic models and closely aligned with segmented regression estimates, reinforcing the robustness of the non-linear association.

### 3.3 Sensitivity and robustness analyses

Robustness checks consistently supported the primary findings. As shown in Supplementary Table 4, quantification of unmeasured confounding using E-values indicated that an unmeasured confounder would need to be associated with both step count and WHO-5 with a risk ratio of at least 1.44 (point estimate) to fully explain away the observed association and at least 1.20 to move the lower bound of the 95% CI to the null. Such magnitudes are unlikely in this context, given the covariates already controlled, suggesting that residual confounding alone is unlikely to account for the observed non-linearity.

The clinical meaningfulness of the dose–response is shown in Supplementary Figure 1, which illustrates the quadratic specification. This model produced an inverted-U curve with a peak at approximately 11,964 steps/day (95% CI: 9,338–14,590), closely aligning with the RCS results.

Additional sensitivity analyses (Supplementary Tables 1–5) confirmed that the results were consistent under alternative model forms, covariate specifications, and trimming or winsorizing strategies. Model fit comparisons (Supplementary Table 6) indicated that the RCS specification provided a slightly better fit than linear or quadratic alternatives, reinforcing its use as the primary model. Finally, post-hoc power analyses (Supplementary Table 7) demonstrated that the study retained adequate sensitivity to detect the non-linear effect (power = 0.786 at *α* = 0.05), supporting the reliability of the inference.

## 4 Discussion

In a relatively homogeneous student sample, we minimized structural confounding from occupation and commuting, enabling a clearer test of the non-linear link between daily steps and SWB. In the RCS model (Figure 1), WHO-5 scores increased steeply with step count up to a data-driven breakpoint of approximately ~8,650 steps/day (from segmented regression), after which further increases produced only minimal gains. The curve reached a statistical plateau with a peak at approximately ~19,300 steps/day (bootstrap-BCa 95% CI: 7,997–17,896; delta method 95% CI: 9,394–14,462); beyond approximately ~8,650 steps/day, the slope was near zero, and the confidence bands largely overlapped, indicating no clinically meaningful improvement at higher levels. The overall non-linearity was significant (*p* < 0.05; Supplementary Table 2), and the segmented model corroborated the same rise-then-plateau pattern. Notably, the maximum predicted contrast relative to 4,000 steps/day did not exceed the prespecified MCID of 10 points.

The pattern aligns with evidence that moderate PA tends to optimize mental health outcomes, whereas ever-higher volumes yield diminishing returns. Large-scale datasets and step-count studies similarly indicate that moving from low activity toward approximately ~5,000–7,000 steps/day is associated with better mental health-related outcomes (Chekroud et al., 2018; Shimura et al., 2022; Bizzozero-Peroni et al., 2024), consistent with our observed plateau pattern and the notion of diminishing returns.

Plausible mechanisms support this non-linear pattern: moderate PA may enhance SWB via stress-buffering and emotion regulation, social engagement, and improved sleep, whereas higher loads—especially late-evening or high-intensity bouts—can exacerbate mood disturbance, HPA-axis strain, injury burden, and sleep disruption. These pathways are coherent with our covariate adjustments (PSQI, PSS-4) and help explain the observed plateau beyond approximately ~8,650 steps/day, where further increases conferred no additional benefit (Stutz et al., 2019; Meeusen et al., 2013; Halson, 2014).

Methodologically, the strengths of this study include objective step measurement, continuous modeling with restricted cubic splines as the primary specification, validation via quadratic and segmented fits, inference with HC3 SEs, and multiple robustness checks (trimming, winsorization, and alternative scales for expenses). Across specifications, the plateau pattern and estimated breakpoint/peak range remained stable (Supplementary Tables 2, 3), suggesting that the results are not artifacts of outliers or scaling choices (Schuster et al., 2022; Muggeo, 2003; Burnham and Anderson, 2004).

Two cautions are warranted. First, peak CIs are relatively wide, reflecting sparse data at very high step counts and greater right-tail uncertainty. Second, the post-peak pattern should not be read as “high steps are harmful”; a more precise interpretation is diminishing returns beyond the optimal zone (Chekroud et al., 2018). As an observational study, residual confounding (e.g., personality traits, prior mental health history, and social support) cannot be ruled out, and generalizability beyond university student’s warrants care.

Practically, the findings support range-based campus guidance: emphasize feasible, moderate targets (e.g., progressing from low steps toward ~5,000–7,000 steps/day already yields benefits) while recognizing individual variability around the plateau onset, beyond which additional steps confer diminishing returns (Chekroud et al., 2018; Shimura et al., 2022; Bizzozero-Peroni et al., 2024). Analytically, we recommend modeling steps as continuous exposure (e.g., RCS, segmented regression, or fractional polynomials) within an information-criterion framework rather than coarse categorization to better capture the underlying curve (Schuster et al., 2022; Muggeo, 2003; Burnham and Anderson, 2004).

### 4.1 Limitations and next steps

As an observational analysis, causality cannot be inferred, and residual confounding may remain; right-tail precision is limited where very high step counts are sparse, particularly in the plateau range. Future studies should replicate across campuses and regions using prospective or quasi-experimental designs, incorporate objective sleep metrics and physiological stress markers, and test mediation by sleep and perceived stress to clarify mechanisms. In addition, the accelerometer sampling frequency and proprietary algorithms of the Huawei Band are not publicly disclosed. This lack of transparency may reduce reproducibility and limit comparability across devices and studies, despite our multiple robustness checks.

## 5 Conclusion

In a large sample of Chinese university students, daily steps and SWB followed a non-linear pattern characterized by an initial rise and subsequent plateau: after covariate adjustment, SWB increased with higher steps up to a breakpoint approximately ~8,650 steps/day and reached a statistical peak of approximately ~19,300 steps/day (bootstrap-BCa 95% CI: 7,997–17,896; delta method 95% CI: 9,394–14,462), beyond which gains leveled off with modest diminishing returns rather than harm. The curve—and its plateau range—was robust across alternative specifications (RCS, segmented regression) and preprocessing choices, supporting a continuous dose–response interpretation.

## Data Availability

The original contributions presented in the study are included in the article/Supplementary material, further inquiries can be directed to the corresponding author/s.
