# Supplementary figures and images for: Inverted-U association between daily steps and WHO-5 in university students: non-linear modeling and robustness checks
Source: Front Behav Neurosci. 2025 Oct 24;19:1693386. doi: 10.3389/fnbeh.2025.1693386 (PMC12592034; doi:10.3389/fnbeh.2025.1693386)

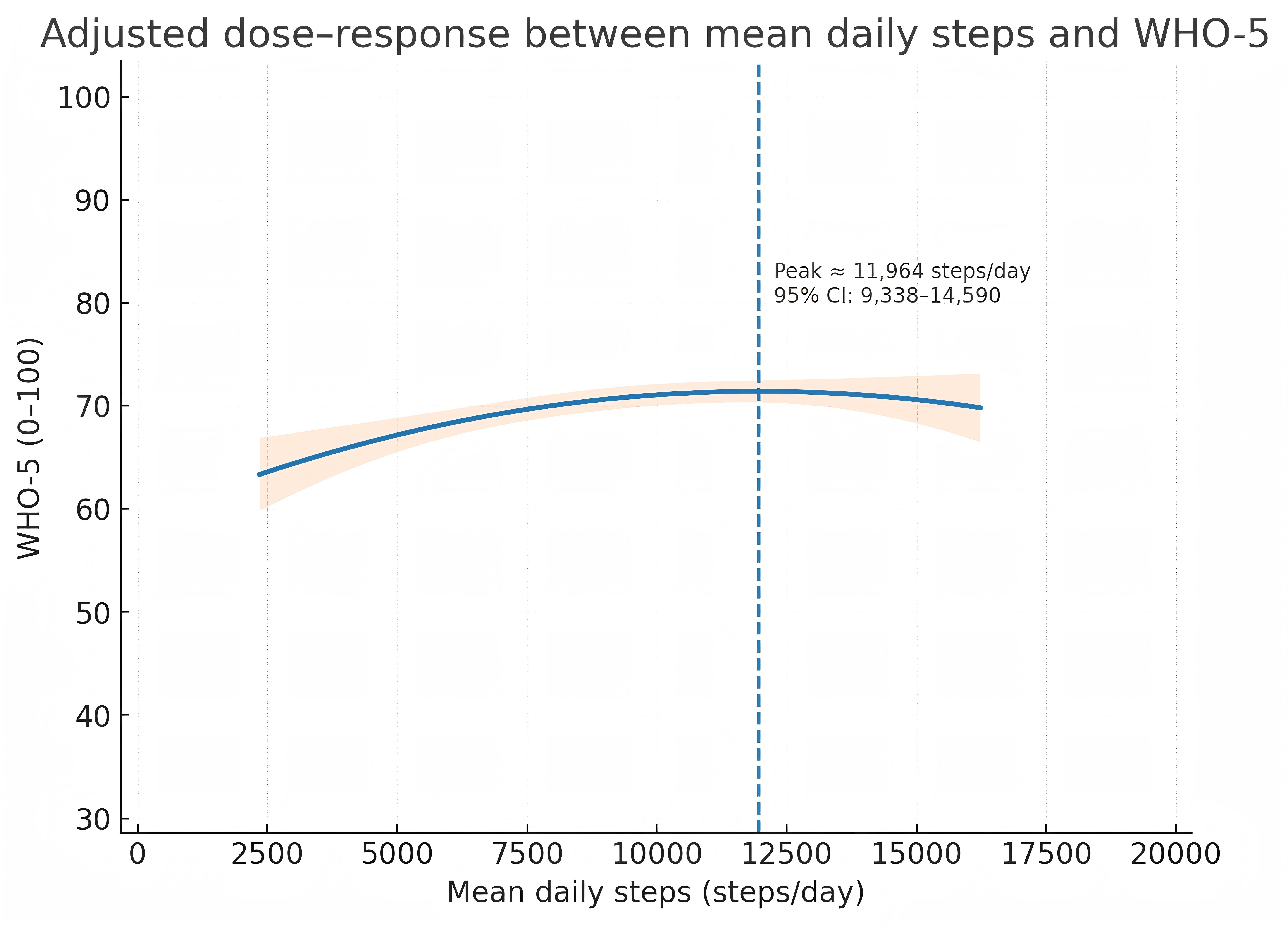

Supplement: Supplementary file 1 [file Image_1.jpeg]
